# The human genome: a multifractal analysis

**DOI:** 10.1186/1471-2164-12-506

**Published:** 2011-10-14

**Authors:** Pedro A Moreno, Patricia E Vélez, Ember Martínez, Luis E Garreta, Néstor Díaz, Siler Amador, Irene Tischer, José M Gutiérrez, Ashwinikumar K Naik, Fabián Tobar, Felipe García

**Affiliations:** 1 Escuela de Ingeniería de Sistemas y Computación, Universidad del Valle, Santiago de Cali, Colombia; 2 Profesora del Departamento de Biología, FACNED, Universidad del Cauca, Popayán, Colombia; 3 Escuela de Ciencias Básicas. Facultad de Salud, Universidad del Valle, Santiago de Cali, Colombia; 4 Departamento de Sistemas, Universidad del Cauca, Popayán, Colombia; 5 Instituto de Física de Cantabria, Universidad de Cantabria-CSIC, Santander, España; 6 Vaatsalya HealthCare Solutions Pvt Ltd, Bangalore, India

## Abstract

**Background:**

Several studies have shown that genomes can be studied via a multifractal formalism. Recently, we used a multifractal approach to study the genetic information content of the *Caenorhabditis elegans *genome. Here we investigate the possibility that the human genome shows a similar behavior to that observed in the nematode.

**Results:**

We report here multifractality in the human genome sequence. This behavior correlates strongly on the presence of Alu elements and to a lesser extent on CpG islands and (G+C) content. In contrast, no or low relationship was found for LINE, MIR, MER, LTRs elements and DNA regions poor in genetic information. Gene function, cluster of orthologous genes, metabolic pathways, and exons tended to increase their frequencies with ranges of multifractality and large gene families were located in genomic regions with varied multifractality. Additionally, a multifractal map and classification for human chromosomes are proposed.

**Conclusions:**

Based on these findings, we propose a descriptive non-linear model for the structure of the human genome, with some biological implications. This model reveals 1) a multifractal regionalization where many regions coexist that are far from equilibrium and 2) this non-linear organization has significant molecular and medical genetic implications for understanding the role of Alu elements in genome stability and structure of the human genome. Given the role of Alu sequences in gene regulation, genetic diseases, human genetic diversity, adaptation and phylogenetic analyses, these quantifications are especially useful.

## Background

The human genome is one of the most complex molecular structures ever seen in nature. Its extraordinary information content has revealed a surprising mosaicims between coding and non-coding sequences [1-4]. This highly regionalized structure introduces complex patterns for understanding the gene structure and repetitive DNA sequence composition and its role in human development, physiology, medicine and phylogeny. The coding regions are defined, in part, by an alternative series of motifs responsible for a variety of functions that take place on the DNA and RNA sequences, such as, gene regulation, RNA transcription, RNA splicing, and DNA methylation. For example, sequencing of the human genome revealed a controversial number of interrupted genes (25,000 - 32,000) with their regulatory sequences [1,2] representing about 2% of the genome. These genes are immersed in a giant sea of different types of non-coding sequences which make up around 98% of the genome. The non-coding regions are characterized by many kinds of repetitive DNA sequences, where almost 10.6% of the human genome consists of Alu sequences, a type of SINE (short interspersed elements) sequence [3]. These elements are not randomly distributed throughout the genome but rather are biased toward gene-rich regions [5]. They can act as insertional mutagens and the vast majority appears to be genetically inert [6]. LINES, MIR, MER, LTRs, DNA transposons, and introns are other kinds of non-coding sequences, which together conform about 86% of the genome. In addition, some of these sequences are overlapped one to another, for example, the CpG islands (CGI), which complicates analysis of the genomic landscape. In turn, each chromosome is characterized by some particular properties of structure and function. Furthermore, the new era of rapid sequencing methods will have available more than one thousand human genome sequences [7], which reveals the genetic variation between different human groups. This knowledge will have a major impact on human health (disease origin), population studies and adaptation, among others. All these structural variations are challenging the inventive of theoretical and experimental scientists to create, develop and apply new approaches to quantify them. These variations allow carrying out studies of comparative genomics aimed at discovering correlations with some life characterizing properties [8,9]. Given that all these genomic variations produce a regionalized genomic landscape in the human genome, we thought fractal geometry could be an appropriate approach to studying how the genetic information content is fragmented.

The methodologies derived from fractal geometry have been a very useful approach to studying the degree of fragmentation (or irregularity) in natural, artificial and statistical structures or processes [10]. Fractal structures are characterized by self-similarity, scaling independence, and a fractal dimension, an exponent obtained from a power or scaling law [11,12]. Thus, power laws are powerful tools for searching self-similar properties in biological structures and processes and for quantifying the scaling properties of information contents.

Few studies have used the fractal approach and power laws to study the whole human genome [13,14]. However, due to the complexity of the human genome, one exponent may not be enough to characterize a complex phenomenon. Multifractal formalism allows using more exponents [15]. In this case, the object of analysis is divided into several fractal sets, each generating a fractal dimension that is then translated into a continuous spectrum of exponents (the so-called singularity spectrum). The multifractality degree (MD) obtained from this continuous spectrum allows measuring the genetic information content. Multifractal systems are common in nature, especially in geophysics. They include fully developed turbulence [16], stock market time series, heartbeat dynamics [17], human gait, and natural luminosity time series, among others. In post-genomics times, multifractal analysis has been a very useful approach to studying problems related with microorganism classification [18,19], distinguishing coding and non-coding sequences [20], studying proteins [21], promoter prediction [22], and - recently - this formalism was used to study human chromosomes [23] and the genetic information content in the *C. elegans *genome [24]. In the latter work, a significant relationship between the structural genetic information content and multifractal parameters was found, which has important biological implications. We thought that applying a similar method could be a valid approach to study the structure of the human genome. In the present paper, we report multifractal analysis from the draft sequence of the human genome.

## Results

**Figure 1:**
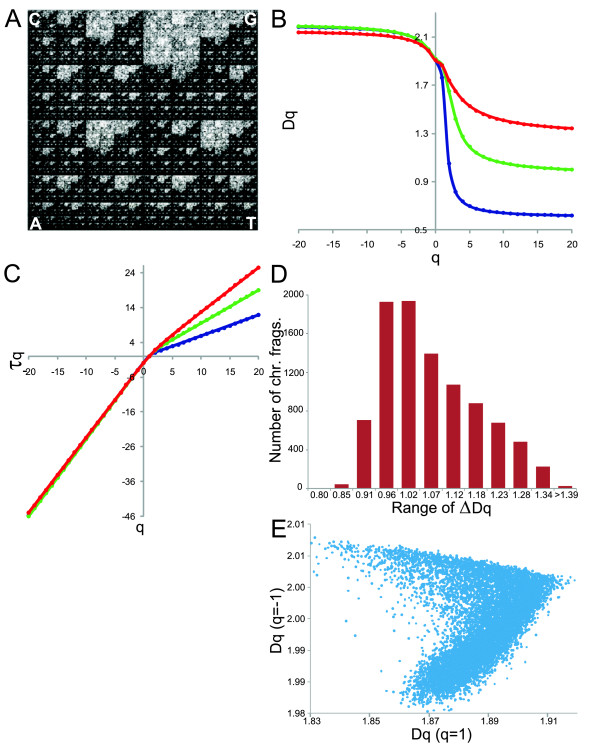
**Analyses of multifractal parameters**: **A: **CGR of an *H. sapiens *chromosome I fragment (~80,000 bp). **B: **Generalized dimension spectra for two chromosome fragments with the highest (blue) and lowest multifractality (red). A medium multifractality is depicted (green) for comparison. **C: **Multifractal spectrum τ(*q*) for the fragments of **B**. **D: **Number of chromosome fragments per RM. **E: **Distribution of 2-D points (*Dq *(*q *= 1), *Dq *(*q *= -1)) of the human genome. *Dq *(*q *= 1) is called the information dimension.

Three approaches were followed to examine multifractality in the human genome from the Chaos Game Representation (CGR) (Figure 1A).

### 1) Multifractal analysis by chromosome fragment

#### 1.1) Analyses of multifractal parameters

The multifractal parameters for 9,379 chromosome fragments were calculated and analyzed (Additional file 1). Initially, the generalized dimension spectrum and MD for all chromosome fragments were determined. The extreme generalized dimension spectra and a medium spectrum are depicted for comparison (Figure 1B). Note that the maximum varies very little due to the fact that negative *q *values are associated with the structure and properties of sparse regions, with few points in the CGR of the human genome. In contrast, the *Dq *minimum varies widely because positive *q *values emphasize regions where the points are dense.

Subsequently, the corresponding scaling exponents τ(*q*) were calculated for each fragment (Additional file 2). The three multifractal spectra τ(*q*) show differences related to each other (Figure 1C). The scaling exponent τ(*q*) can reveal aspects of chromosome fragment structure. Monofractal behavior would correspond to a straight line for τ(*q*); for multifractal behavior, τ(*q*) is nonlinear. The changing curvature for the data for the chromosome fragments indicates multifractality. In contrast, τ(*q*) tends to be linear for that chromosome fragment with the lowest multifractality, indicating partial loss of multifractality.

Using the whole data set for each chromosome we calculated the MD from each generalized dimension spectrum. Thus, the degree of multifractality for all chromosome fragments goes from ~0.79 to 1.56 with an average of 1.042 and median of 1.018 (Additional file 1). Analysis by range of multifractality (RM) reveals that the multifractal behavior for the whole data set is biased toward low multifractal values, as expected (Figure 1D).

Next, we used a discrimination method based on 2-D distributions to study the information dimension for all chromosome fragments. The data show two different informational patterns (similar to a > symbol), one with high information content (Figure 1E, dots on top) and the other with low and medium information content (Figure 1E, dots on bottom) being the occurrence the latter more numerous in data than the former. We hypothesize that these behaviors are related with some molecular parameter, which is analyzed in the following section.

#### 1.2) Analyses of molecular parameters

**Figure 2:**
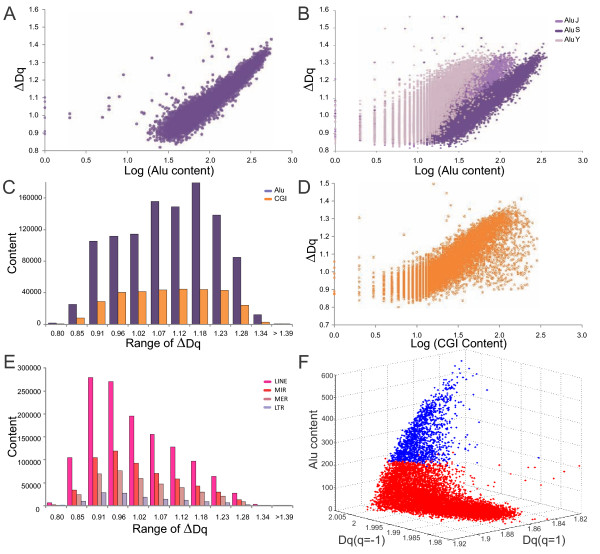
**Analyses of molecular parameters**: Relationships between the MD *versus ***A: **Alu content, R^2 ^~0.86, *p *< 0.05 and **B: **Alu subfamilies: Alu-S (R^2 ^~0.84, *p *< 0.05), Alu-J (R^2 ^~0.7), and Alu-Y (R^2 ^~0.52). **C: **Alu content per range of Δ*Dq*. **D: **Multifractality *versus *log (CGI), R^2 ^~0.64, *p *< 0.05. **E: **LINE, MIR, MER and LTR contents per RM. **F: **Distribution of 3-D points (*Dq *(*q *= 1), *Dq *(*q *= -1), Alu content) of the human genome. We used a cut point ≥ 217.9 Alus (blue dots) according to paragraph 1.4.

The annotated contents of coding and non-coding sequences for each fragment were determinated (Additional file 1). These counts were similar to those reported by other studies [1,2] suggesting that our results are consistent at chromosome level. We hypothesize that these multifractal behaviors might be explained by different repetitive DNA contents in the human genome, similar to the results found in *C. elegans *[24]. Therefore, we examine several molecular density parameters against the MD. We especially focused on the Alu sequence content, given its high polymorphism. We observe 1,078,720 Alu sequences which is equivalent to about 10.58% of the human genome where chromosome fragments contain 0-563 Alu sequences with an average of 115 Alus, *i.e*., one Alu element for about every 2,600 bp of genomic DNA. We demonstrated how strong the relationship between the MD and Alu content is (Figure 2A).

This relationship was assessed in terms of Alu families and the Alu-S was found to be more correlated than the other Alu families (Figure 2B). Furthermore, the Alu contents (in conjunction with CGI) are biased toward high multifractal ranges suggesting the significant role of these sequences in determining the non-linearity in the human genome (Figure 2C).

When sequencing the human genome, a strong relationship between Alu and CGI contents [1,2] also became evident. We observe that CGI have a lower multifractal relationship than that found for the Alu elements (Figure 2D). However, when both parameters are combined a significant fit was obtained (R^2 ^= 0.85, *p *< 0.05). Other molecular parameters such as gene density, exons, introns, LINE, MIR, MER, and LTRs did not show a significant fit by a simple linear regression. However, when all repetitive elements (Alu, LINE, MIR, MER, and LTRs) are taken into account the R^2 ^~0.57. Thus, among the studied genomic features, Alu has the highest correlation with multifractal degree.

Multivariate analyses of Δ*Dq *versus all variables (Alu, G+C, CGI, LINE, MIR, MER, LTRs, nCoding, nNonCoding, exons, genes, and SNPs) per chromosome were carried out and for each case the most relevant variables explaining Δ*Dq *were selected. The most frequently used variables are (G+C), Alu, CGI, which are significant in 23, 23, 21 cases of the 24 chrs. (Additional file 3). CGI coefficients in all regressions are negative, probably compensating the high positive (G+C) coefficients, given that (G+C) and CGI are strongly correlated (R ~0.805). Positioning Alus among the most relevant variables confirms our prior analyses based on 1 and 2 dimensional regression. In the same way, we analyzed Δ*Dq *for the whole genome, obtaining again (G+C), Alu, CGI as the most relevant variables explaining Δ*Dq *(Additional file 4). Moreover, when the long interspersed repeats are analyzed by RM they tend to be located on low and medium multifractality (LMM) ranges (Figure 2E).

Given that the information dimension studied takes a form of > symbol (Figure 1E), we studied its behavior using a discrimination method based on 3-D distributions. In this analysis, the high information content is related to Alu content, whereas the low and medium information contents are rather related to low Alu contents and other genomic structures (Figure 2F).

#### 1.3) Multifractal map of the human genome

**Figure 3:**
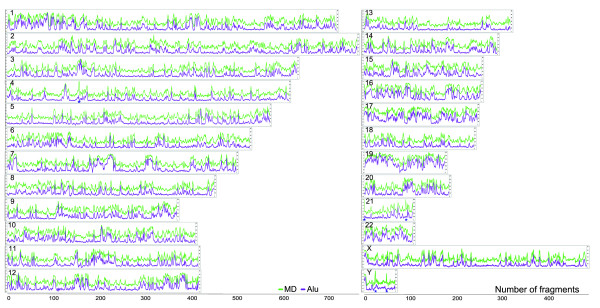
**Multifractal map of the human genome**. Overview between the MD (green) and Alu density (purple) across the human chromosomes. (*): VSTRs.

We examined the multifractality and Alu content across the genomic landscape to map these relationships in each human chromosome. The analysis reveals how similarly these two variables behave. This is particularly clear when the determination coefficient for the linear regression is calculated for all chromosomes (Figure 3, Additional file 5). All R^2 ^oscillate between ~0.78 and 0.92, with the exception of chromosomes Y, 21, 19, X, and 11. The apparent low correlation (0.24 ≤ R^2 ^≤ 0.76) of these chromosomes can be explained by the presence of some atypical chromosomic fragments: they may contain some kind of repeat (chrs. 4, 21, and Y) or present a lack of Alu contents (chrs. 11, 12, and 19). Once these fragments (nine in total) are removed from the analysis, the R^2 ^for all chromosomes improve significantly (0.78 ≤ R^2 ^≤ 0.92, *p *< 0.05), including chromosome Y (R^2 ^= 0.52). The chr. 17 has the highest determination coefficient between multifractality and Alu content with chromosome Y having the lowest. With the exception of the atypical chr. Y, these results indicate that multifractality in each human chromosome is dependent on the content of repetitive DNA - type Alu-. Additionally, other determination coefficients for several molecular parameters were calculated in this study (Additional file 5). Thus, among the studied molecular parameters, Alu shows the highest correlation with MD. A multivariate regression analysis also showed a similar result (Additional file 3).

Given that other repetitive elements could contribute to increase the local multifractality, five chromosome fragments with a low number of Alus and high MD in chromosomes 4, 21, and Y called our attention (Figure 3, asterisks, Additional file 5). We analyzed these sequences and found many variable short repeats in tandem (VSRTs) (Additional file 6). Thus, the presence of these repeats increases local multifractality but reduces the entire chromosome multifractality for these chromosomes, as mentioned before.

#### 1.4) Chromosomal location of the most multifractal chromosome fragments

**Figure 4:**
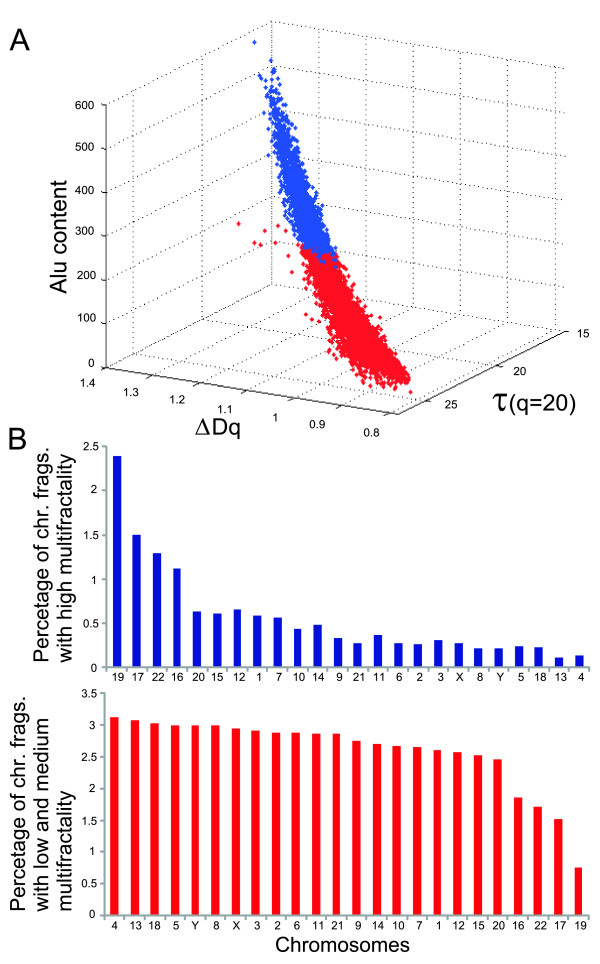
**Genomic location of the most multifractal chromosome fragments**: **A: **Discrimination method based on three parameters. Each chromosome fragment dataset is characterized by three quantities. The first quantity (*x*-axis) is the MD for each chromosomic fragment. The second quantity (*y*-axis) is the density of Alu content of the chromosomic fragments. The third quantity (*z*-axis) is the correlation coefficient τ(*q*). Blue color indicates those fragments with Δ*Dq *≥ 1.159 and Alu contents ≥ 217.9. **B: **Above, distribution for chromosome fragments with high multifractality and below, for fragments with LMM.

Human genome sequencing revealed that chromosomes 19, 16, 17, and 22 are richer in genes, CGI, and Alu elements [1]. Based on averages for Δ*Dq *and Alus for these chromosomes we defined a threshold for chromosome fragments as Δ*Dq *≥ 1.159 and Alu contents ≥ 217.9 (Additional file 7). This allows to separate chromosome fragments with the highest multifractality from those with LMM (Additional file 8). A discrimination method based on distributions of 3-D points shows how both groups of chromosome fragments can be easily differentiated (Figure 4A). The plot reveals that the highest multifractality and Alu contents are observed in 1,292 fragments suggesting the existence of an abundant number of multifractal regions in the human genome with an average multifractality around 1.24 and an average of ~305 Alus. As expected, many fragments (~29%) are located on chromosomes 19, 17, 16, and 22, respectively (Figure 4B, above). Similar results were obtained by using a 3-D plot with MD-Alu-*Dq*(*q *= 1) (data not shown). Chromosome fragments with LMM and low Alu contents, in contrast, are situated mainly on the other chromosomes (~86.2%), being chromosomes 4, 13, 18, 5, and Y those with the lowest multifractality (Figure 4B, below), an average multifractality of 1.0, and average Alu of ~79.

#### 1.5) Analyses by gene function, gene family, and gene length

**Figure 5:**
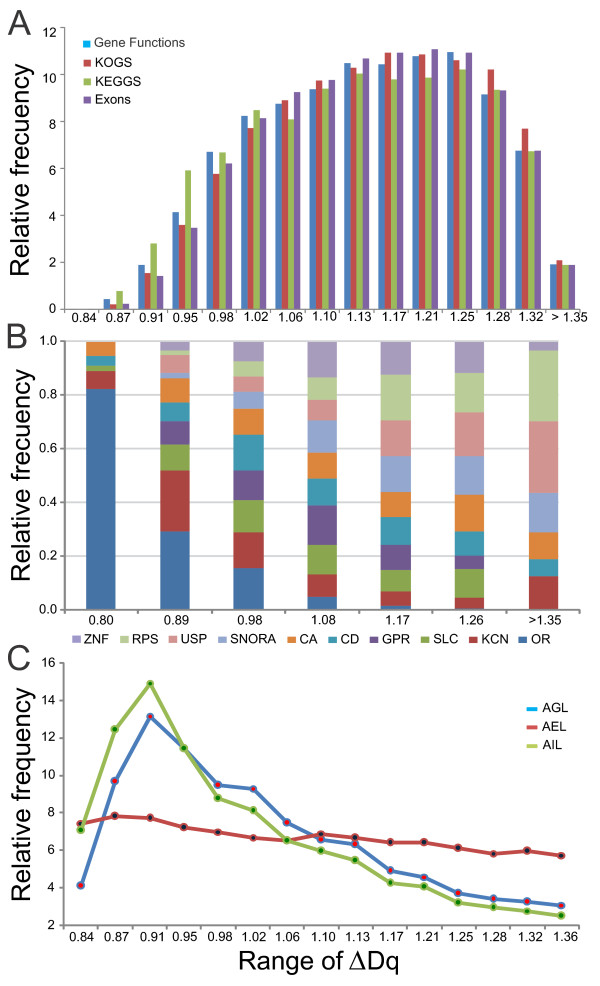
**Distributions by gene function, gene family, and gene length**: **A: **Gene functional distributions per RM. These distributions are strongly significant up to 80% of the ranges. **B: **Percentage of gene families per RM. Gene families: CA: Carbonic anhydrase, CD: cluster of differentiation, GPR: G protein-coupled receptors, KCN: potassium channels, OR: olfactory receptor, RPS: ribosomal proteins, SLC: solute carrier, SNORA: small nucleolar RNA, USP: ubiquitin-specific peptidases, ZNF: Zinc fingers, C2H2-type. **C: **Degree of gene fragmentation per RM. AGL: average of gene length, R^2 ^~0.55. AEL: average of exon length, R^2 ^~0.91. AIL: average of intron length, R^2 ^~0.74.

One would expect to find other molecular characteristics of the gene associated with the multifractality; hence other related molecular parameters were examined. Several biased distributions toward high ranges of multifractality for gene functions, cluster of orthologous genes (KOGS), metabolic pathways (KEGGs), and number of exons were found (Figure 5A, Additional file 9). We only found gene function information for 5,823 chromosome fragments with an average multifractality degree (AMD) of 1.126 and median of 1.132. For example, many genes for the cell division cycle lie on chromosome fragments with an AMD of 1.203; many genes of the major histocompatibility complex, classes I and II are situated on fragments with AMD = 1.06; and many members of the melanoma antigen family lie on fragments with AMD = 0.96 to mention a few.

To gain further insights into the gene function, we focused on about 208 human gene families, consisting of 4,614 genes [25,26]. We asked about the multifractal genomic context for these gene families. The distributions obtained show three different multifractal behaviors (Figure 5B): low-skewed (for OR, KCN, HLA, IFN, KRT, CDH, and RGS), high-skewed (for ZNF, SNORA, USP, RPS, SNORD, GTF, DHX, ALOX, and UBE2), and "medium" for most gene families. Other gene families can be placed within some of these categories (Additional file 10).

When multifractality is related to the information content (for example, number of exons, Figure 5A), it is expected that the more genetic information exists, the greater is the extent of genetic information fragmentation. To verify this assumption we looked for the average lengths of genes, exons, and introns in relation to the RM. The three corresponding distributions show how the average lengths decrease as multifractality increases (Figure 5C, Additional file 11). Another approach to validate this assumption is to observe the number of information units (IU) (exons plus introns) per RM. Here, the distribution shows that the number of IUs increases when the RM increases (data not shown).

### 2) Multifractal analysis by chromosome

**Figure 6:**
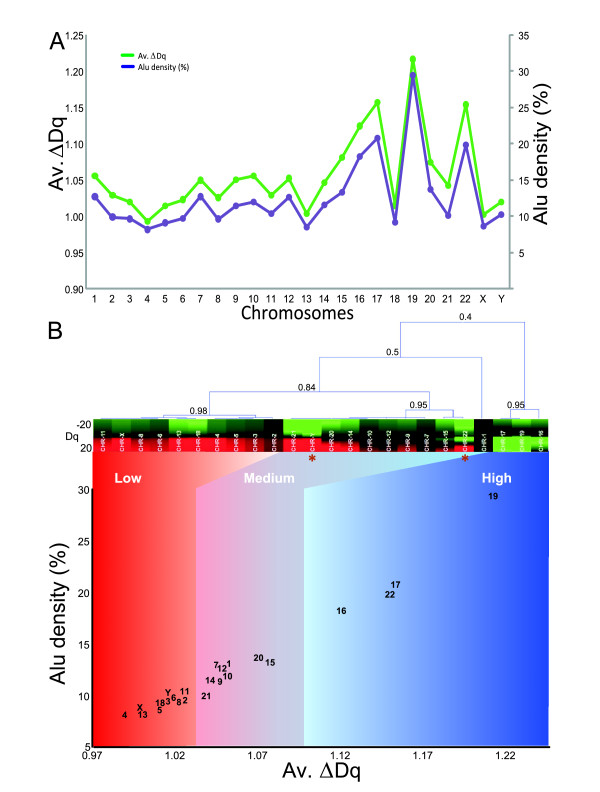
**Multifractal classification for the human chromosomes**: **A: **Distributions of the average degree of multifractality (Av. Δ*Dq*) and Alu content per chromosome. **B: **Discrimination method based on multifractal formalism in a distribution of two-dimensional points, R^2 ^~0.967, *p *< 0.05. On top: hierarchical clustering for the averaged multifractal parameters by chromosome between *Dq*(-20, 20) (color scale bar is indicated). Minimum similarities are indicated near nodes and the asterisks show the only two exceptions found.

We next explored the multifractal behavior of each chromosome. We found that the AMD and Alu content profiles have very similar behaviors (Figure 6A). This is particularly evident when observing how well these two variables fit (Figure 6B, Additional file 12). Following the linear regression line, three groups of chromosomes can be distinguished (by visual inspection): a first group where chromosomes 19, 17, 22, and 16 exhibit the highest multifractality (and the highest Alu contents), a second group consist of chromosomes 15, 20, 1, 10, 12, 9, 7, 14, and 21 with medium multifractality, and a third group of chromosomes 2, 11, 8, 6, Y, 3, 18, 5, 13, X, and 4 with the lowest multifractality, respectively. A similar analysis showed that the CGI were also highly correlated with the AMD (R^2 ^~0.86, *p *< 0.05) (Additional file 13).

We asked whether this subjective classification could be obtained by hierarchical clustering of the complete data set of averaged multifractal parameters, using multifractality as a similarity measure (Additional file 14). The clustering process classified the chromosomes into three multifractality groups (on top of Figure 6B), with among group similarities of 0.84 and 0.4, respectively: low, medium and high confirming (in part) the visual observation. Nearly all chromosomes (92%) lie on the consecutive, visually identified low, medium and high sections on the regression line. The only exceptions are chromosomes 22 and Y, which are placed in other groups.

### 3) Multifractal analysis by average of chromosome regions

**Figure 7:**
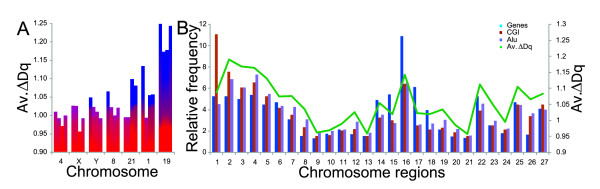
**Multifractality per average of chromosome región**: **A: **Multifractal distribution per chromosome, where each chr. region (each bar) has an equal length. Blue color represents those chromosome regions with high averaged multifractality (Δ*Dq *> 1.04). Degraded blue-red color depicts medium multifractality (Δ*Dq *≤ 1.04). Red color: low multifractality. **B: **Correspondence between averages of gene, CGI (R^2 ^~0.62, *p *< 0.05) and Alu (R^2 ^~0.95, *p *< 0.05) contents versus averaged multifractality across the human chromosome 1.

Analysis by chromosome region proved to be a valid approach to study the genetic information content in the *C. elegans *genome [24]. Here, we applied the same approach to analyze several characteristics of the human genome. It is known that chromosome 21, involved in Down syndrome, shows a degree of asymmetric regionalization in the distribution of the Alu elements (Figure 3) [1,2]. We hypothesized that one part of chromosome 21 should have low multifractality and the other one high multifractality. Indeed, the data show that the first 50% of chromosome 21 is of low multifractality (< 1.0), whereas the other 50% has a higher multifractality (> 1.08) (Figure 7A, Additional file 15).

Other relevant characteristics are observed in some chromosome bands and arms (Figure 7A). For example, the X chromosome, involved in X chromosome inactivation (XCI), which is rich in LINE1 elements and poor in Alu sequences showed a 0.95 ≤ Δ*Dq *≤ 1.027 (Additional file 16). The Y chromosome has two particular regions to the Yp and Yq ends, the pseudoautosomal region and the palindromic region, respectively [1]. We thought that the palindromic regions should have low multifractality because of their symmetric structure. We found, in fact, that this region has lowered non-linearity. Moreover, recombination rates in chromosome 8 tend to be much higher in distal regions (around 20 Mb) [1] and the analysis showed medium non-linearity at this region as expected (Additional file 17). Regarding chromosome 1, rich in Alu sequences in one of its arms [27], we found significantly high multifractality (~1.13) at this region; in contrast, the other three regions have a Δ*Dq *≤ 1.058 (Additional file 18). Similar situations can be analyzed for other chromosomes. As two opposing references we use chromosomes 4 and 19 for comparison (Additional file 19).

Antibodies to histone modifications previously linked to active transcription, showed close correspondence to regions rich in genes and CGI in human methaphase epigenome [28]. We analyzed chr. 1 and found that CGI profiles correspond well to multifractality (Figure 7B, Additional file 20).

### Discussion

We discovered a strong relationship between the multifractal parameters and part of the genetic information coded by the human genome.

### Initially, the multifractality in human genome was found strongly dependent on the Alu contents

Herein, thousands of chromosome fragments with multifractality ranging from low to high values were analyzed (Figure 1A-C). For all chromosome fragments, *τ*(*q*) is a nonlinear function (Figure 1C), indicating that the molecular structure of the chromosome fragments has a multifractal behavior. However, in many chromosome fragments, *τ*(*q*) tends to be close to linear behavior, especially for *τ*(*q≥*2), indicating partial loss of multifractality. These results suggest that nucleotide fluctuations are less anti-correlated in many chromosome fragments. In fact, the fragment distribution is biased toward low and medium multifractal values (Figure 1D), suggesting that the human genome has a large number of regularly arranged elements, highly periodic and not very polymorphic. This is not surprising because the human genome has about 98.9% of non-coding sequences with a complex composition given by introns and intergenic regions. That is, at least 55% of this information is poorly polymorphic given that these regions mainly consist of introns, LINEs (especially L1), LTRs and DNA transposons [1,2]. In contrast, the human genome also has a significant number of chromosome fragments with high multifractality (Figure 1D). That means these regions should be rich in specific types of sequences that are highly polymorphic and organized in a large number of possible combinations. When the information dimension was analyzed a dual informational behavior confirmed such assumption (Figure 1E). Indeed, the multifractality was found to be strongly correlated with the Alu content (Figure 2A), which became visible when plotted against the information dimension (Figure 2F). This result is very significant given that the Alu family is highly polymorphic [29,30] and in a 300 kb chromosome fragment one can find Alu elements in many combinations in up to 50% of its length. The Alu elements are not identical and can be classified into three major families: Alu-J, Alu-S and Alu-Y representing the oldest, intermediate, and youngest Alus, respectively and each family is divided into one or more levels of subfamilies [31]. In total, ~45 subfamilies encompass the complete Alu family. We found that multifractality was mainly dependent on the Alu-S contents (Figure 2B), especially the Alu-Sx, an expected result since these sequences are the most abundant Alu members in the human genome [1]. Analysis via RM confirmed that the Alu sequences tend to be located toward medium and high ranges of multifractality (Figure 2C) because of the high Alu content in the human genome.

The CGI showed a moderate relationship with the multifractality (Figure 2C, D), which might be because more than 95% of CGI are less than 1,800 pb long [1]. Genes, exons, introns, LINES, MIR, MER and LTR contents did not show any significant relationship with the multifractality because most of these sequences have a low number of members, are large and have few polymorphisms. For example, LINE elements are ~6 kb long, more numerous than Alus and consist of four families, being LINE-1 the most abundant family (~17%) in the genome [32], and their density pattern is quite uniform for most chromosomes [1]. Thus, the combination of number of members, size and polymorphism seem to be determining characteristics for multifractality changes. The earlier mentioned abundant number of polymorphic Alu sequences confirms the relation between these characteristics and multifractality. In fact, an in silico comparative genomics study between public and Celera versions of human genome sequences identifies several hundred new Alu insertion polymorphisms, showing that these elements are highly polymorphics [31]. A similar behavior is found in *C. elegans *where the TTAGGC repeat is abundant in number and combinations within the flanking sequences [24].

**Subsequently, we elaborated a multifractal map of the human genome **(Figure 3), which shows MD and Alu density along the human chromosomes. The map reveals that the human chromosomes contain many significant correlation structures for Alu-rich regions. Thus, the high contents of Alu account for the high aperiodicity and genetic variability of many chromosome sections. A similar result in *C. elegans *reported changes in multifractality related to a specific type of repetitive DNA [24]. Additionally, the correlations for CGI are lower but significant. However, no significant correspondence was found in regions poor in Alu sequences and rich in LINE, MIR, MER and LTR sequences. Not all multifractality is due to the Alu contents, many VSTRs can also contribute to increasing local multifractality (Figure 3, asterisks). We found very poor correspondence to the number of genes perhaps due to their low frequency. These results, taken together, indicate that the observed multifractality is primarily related to nonlinear distributions for those chromosome fragments which are rich in Alu sequences, next for those with high CGI content and in few instances, for those with high VSRT contents.

### Hundreds of highly multifractal chromosome fragments mapped in chromosomes rich in genetic information

There were a large number of chromosome fragments with very high multifractality (Figure 4A), mainly located on chromosomes 19, 17, 22, and 16 (Figure 4B, above). All of these chromosome sections, so we suggest, generate a mosaic of regions locating the genetic information far from equilibrium [17,24], which could be interpreted both, as a protector "shield" for the human genome against environmental fluctuations and as "genomic attractors" to maintain many components, functions and processes under a "deterministic" genomic control. In contrast, the same analysis also identified thousands of LMM chromosome fragments (Figure 2C) with low Alu content (Figure 4B, below) and perhaps prone to being affected by the environment. This result might be interpreted as some genome sections with low nonlinearity that might have high genetic instability associated with some particular (structural or functional) gene property.

### Several gene characteristics are related to multifractality

This is not striking since three-fourths of all genes in the genome are associated with Alus (Figure 5A) [30]. Therefore, some gene families tend to be located preferentially within a multifractal genomic context (Figure 5B). For example, the hOR gene family lies mainly on a low multifractal genomic context. This is due to this family has a very periodic and repetitive structure. It is known that the OR gene family has about 390 active members which were propagated on the genome by gene duplication. Hence they share a high homology due to their high structural homogeneity and possess many clusters of regular characteristics; nonetheless, their functional expression depends on a complex interplay between regulatory sequences and the environment [33]. A similar behavior is observed in the KCN gene family, responsible for building potassium channels for cell communication. In contrast, the ZNF gene family, which codes for regulatory proteins and is, therefore, involved in many cellular functions, is located in a medium and high multifractal genomic context. For example, the ZEB2 protein involved in a chemical signaling pathway regulates early growth and development and obeys a pre-determinate genetic program. In addition, these genes have a high structural inhomogeneity and many irregular characteristics. Similar inferences might apply for the RPS gene family, which codes for highly conserved proteins for the ribosome, for the SNORA machinery involved in the nuclear splicing and for USPs that help to control the levels of many proteins in the cell [26]. This seems to suggest that the low multifractal genetic context might be related to information inputs from environmental processes, and the high one to inputs from deterministic processes. Thus, a few gene families in the human genome might be subjected to two types of information (or stimulus) inputs, while most gene families seem to be subjected to a complex regulatory interplay between epigenetic and genetic controls.

On the other hand, the degree of gene fragmentation by RM (Figure 5C) behaves according to the multifractal theory: multifractality increases when the length of exons and introns in the human genome decreases and the number of IUs per interrupted gene increases with multifractality, as expected.

**The multifractal approach per chromosome permitted classifying the human chromosomes. **This analysisvalidated the strong relationship to the Alu elements (Figure 6) we found especially for chromosomes 19, 17, 22, and 16, which are rich in genetic information content [1,2]. Particularly chromosome 19 is by far the most multifractal chromosome and has the highest gene density of the whole genome. It is also unusual with respect to its density of repeat sequences. In fact, nearly 55% of this chromosome consists of repetitive elements, whereas chromosomes 6, 7, 14, 20, 21 and 22 all have repeat contents ranging from 40% to 46% (the genome average is 44.8%). This difference is due mainly to an unusually high content of SINEs in chromosome 19 [1]. In contrast, chromosomes 13, X, and 4 have the lowest multifractality because their Alu content is lower than the autosomal average, they have low gene density. Some of these chromosomes have very large "gene deserts" and the CGI and LINE contents are the highest percentage among all autosomes [1,30]. A similar behavior can be observed for chromosomes 19, 17, and 4, as reported in a recent multifractal analysis [23].

Our analysis permits classifying human chromosomes into three multifractality groups suggesting that the chromosome molecular structure might be organized as a system operating far from equilibrium [24] (Figure 6B). Thus, those chromosomes with low multifractality might be closer to equilibrium and have greater genetic instability. If so, this would explain, why some chromosomes would be involved in some genomic disorders (structural and numerical chromosome alterations)[34]. For example, some microdeletion syndromes have been reported for chr. 4: Wolf-Hirschhorn syndrome, chr. 5: Cri du chat syndrome and chr. 15: Angelman and Prader-Willi syndromes. Some aneuploids can be present in chr. 8: Syndrome of Warkany, chr. 13: Patau syndrome, chr. 18: Edward syndrome, chr. 21: Down syndrome, chr. X: Turner syndrome (XO), Klinefelter syndrome (XXY), triple X syndrome and other tetra and pentaploids of chr. X. For chr. Y: XYY syndrome and Turner syndrome. With the exception of chromosomes 21 and Y, all were classified as chromosomes with low multifractality and are more susceptible to genetic damages or a wrong meiotic segregation.

### The multifractal approach by chromosome region reveals different genomic scenarios (Figure 7A)

For instance, 21 chromosome regions with low multifractality might promote genetic instability during meiotic segregation in Down syndrome. Similar behaviors might arise for chromosomes X and Y to explain XCI and sex determination. For example, the most remarkable enrichment of repetitive sequences obtained for L1, which accounts for 29% of the X chromosome sequence compared to the average of only 17% [1]. Some studies have reported significant association between L1 and coverage and inactivation, and others have refuted this result [35]. However, the low multifractality, especially at the third region (AMD ~0.96) may be prone to XCI. With regard to chromosome Y, the pseudoautosomal region is more stable, while the palindromic (more periodic) region is unstable and more prone to producing some genetic disorder such as the mixed gonadal dysgenesis and infertility [1,34]. On the contrary, the 8p region in which a vast section of ~15 Mb has a strikingly high mutation rate lay on a medium multifractality region [36]. Similar behavior can be inferred in the *C. elegans *chromosome arms, rich in mutation rates [24].

A similar approach showed that the CGI and Alus correspond well to multifractality (Figure 7B). This result is significant because of the role that CGI play in heritability of epigenetic states during the active transcription or modifications associated with active chromatin [28].

### Finally, we propose a descriptive, non linear model for the function and organization of the human genome (Figure 8)

**Figure 8:**
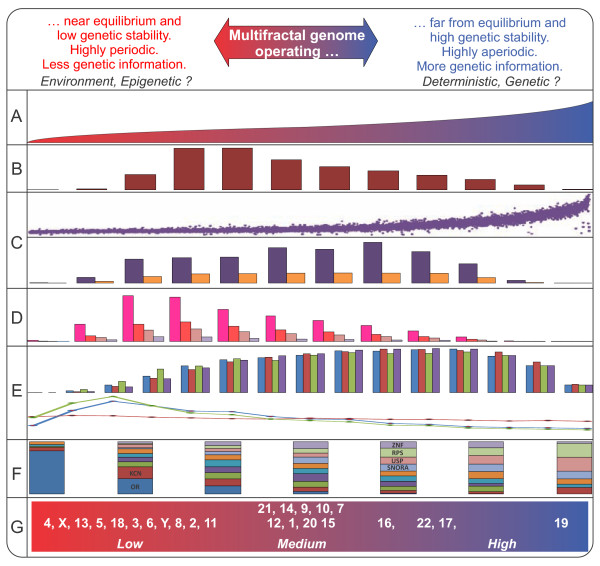
**Summary diagram: a conceptual non linear model for the human genome**: From left to right multifractality increases. In A: multifractality profile for 9,379 chromosome fragments (from 0.79 to 1.56). In B: Figure 1D. In C above: Alu content profile for 9,379 chromosome fragments and below Figure 2C. In D: Figure 2E. In E: Figure 5A, C. In F: Figure 5B. In G: Figure 6B.

Firstly, several studies have suggested that multifractal systems might be organized as systems operating far from equilibrium [16,17,24]. Thus, the detection of a multifractal scaling in the human genome structure suggests that its molecular structure might be organized as a system operating far from equilibrium, meaning that no variable describing the state of the system shows a regular repetition of values. The high multifractality which strongly depends on Alu contents (and upon CGI to a lesser degree) and is located mainly in highly aperiodic regions, takes the chromosome away from equilibrium giving greater genetic stability, protection, and attraction of mutations (Figures 2A-C, 3F, 3, and 8C). Thus, hundreds of regions in the human genome might have a high genetic stability (Figures 1B-E, and 8A, B) and the most important genetic information of the human genome (genes) would be safeguarded from environmental fluctuations. It is because Alu elements (and CGI) are biased toward gene-rich regions [5]. Furthermore, it is well known that the Alu elements are highly polymorphic [29] or highly aperiodic and that a marked reduction of Alus is located within the interrupted genes, especially in exons [6]. Hence, a great number of mutations fall into the flanking regions of the coding sequences [37] and Alu elements become effectors of gene transcription by providing new enhancers, promoters and polyadenylation signals to many genes [38]. Based on these findings and those found in *C. elegans *[24], it seems that the non-linearity might be located on highly polymorphic genetic units that are distributed in many combinations through the genome. If so, we inquired on how these sequences have come to exist. Possibly, this might be explained by the fact that the multifractal scaling in the human genome appears to be located on fractal structures, which are mathematically created (in a deterministic way) by superposition of seed sequences [23]. So these seeds may have been the Alu sequences, which might have increased in number by retrotransposition, a process involving the insertion of reverse transcribed DNAs of Alu-derived transcripts back into the genome, apparently by hijacking the LINE-1 retrotransposition machinery [31]. Thus, multifractality may have occurred extensively in the past by the apparent "over-transposition" of different functional units (Alus, CGI) carried by each DNA sequence. Nowadays, it is hypothesized that the majority of transposable elements have been silenced perhaps by some repressive mechanism [39] to protect the genome. However, our results suggest that the Alu elements may themselves be responsible for genetic stability and protection to the genome. Thus, the human multifractal map developed here provides a tool to identify regions that are rich in genetic information and genome stability.

Secondly, there is a strong tendency to increase genetic information content when multifractality increases and to increase gene fragmentation when multifractality increases. These results are consistent with what the multifractal theory predicts (Figures 5A, C, and 8E). Thus, the human genome seems to be made by many information units (interrupted genes, Alus and CGIs) with different degrees of fragmentation (or size) that account for the aperiodic scaling of short and long range correlations found by other authors [14].

Thirdly, a multifractal genomic context seems to be a significant requirement for the functional and structural organization of thousands of genes and many gene families, *i.e*., a low multifractal context seems to be necessary for many sequences (generated by gene duplication and periodicy) to interact with environmental signals, while a high multifractal context (aperiodic) seems to be prone (or a "genomic attractor") to many genes; and some (very aperiodic) gene families are involved in deterministic and genetic processes (Figures 5A, B, and 8E, F). Thus, the highly multifractal regions would be a guaranty to maintain a deterministic regulation control in the genome [24], although most of the human genome sequences can be subjected to a complex epigenetic and genetic control as observed when the human epigenome due to the CGI contents is related to multifractality [28].

Fourthly, the human chromosome classification and some chromosomic region assays may have some medical implications. That is, the structure of low non-linearity exhibited for some chromosomes (or chr. regions) might imply an environmental predisposition to be sensible targets for structural and numerical chromosomic alterations (Figures 6, 7, and 8G). In fact, the loss of non-linearity is associated with failure or alterations of many vital systems close to equilibrium [17,40,41]. Additionally, the sex chromosomes must have low multifractality to maintain the sexual dimorphism and likely the XCI.

All these fractal and biological arguments might explain why the Alu elements are shaping the human genome in nonlinear manner. We believe that applying comparative multifractal genomics among many human genomes and other model organisms can help to respond to how the genome came to exist.

## Conclusions

We report evidence for multifractality in the human genome. We identified thousands of chromosome fragments with low, medium and high multifractality, which can be translated in terms of variable genetic stability. Using these fragments we demonstrated -by different approaches- that changes in multifractality depend strongly on changes in contents of Alu sequences. The generated multifractal map of the human genome allows discussing the multifractal context in which thousands of genes and repetitive sequences lie. Thus, the Alu elements (and CGI) are non-linearity shapers and protectors of the genetic information of the human genome.

Likewise, the averaged multifractality permitted analyzing chromosome regions and classifying human chromosomes into three groups. This non-linear classification has significant medical implications because it is able to explain some chromosomal disorders, among other genomic particularities.

All of these findings help to propose a useful and integrative conceptual non-linear model to discuss and quantify the structural variation and nonlinear organization of the human genome.

## Methods

### Databases, sequences, and multifractal approaches

The Hs_refseq human genome sequence build 36.2 was downloaded from the NCBI web site [42]. Three multifractal approaches were followed in this study: 1) By chromosome fragment, 2) by chromosome, 3) by average of chromosome regions. In the first approach, we tested several fragment sizes of DNA sequence and we found 300 kb was an adequate length to be analyzed. This selection was based on several criteria such as percentage of discarded genome, average gene size, gene family, genetic and multifractal context, and scale independence for chromosome fragment size (Data not shown). Nevertheless, other sizes could have been taken into account. Subsequently, the contig order for each chromosome was defined according to the contig files at the 36.2 version and each contig was divided into fragments of 300 kb. That resulted in 9,389 fragments, representing 2,816,700 kb. It is about 98.6% of the whole human genome, discarding about 1.4% of the genome. Another ten chromosome fragments were removed from the analysis, because of an excessive number of Ns and lack of annotation, leaving 9,379 chromosome fragments (Additional file 1). By using these fragments, five types of analyses were implemented: analyses of multifractal parameters, analyses of molecular parameters, multifractal map of the human genome, chromosomal location of the most multifractal chromosome fragments, and analyses by gene function, gene family, and gene length. In the second approach, the resulting fragments per chromosome were averaged for multifractality to obtain a measure for each chromosome. In the third approach some chromosomes with some structural particularities were studied. Here, the resulting fragments per chromosome from the first approach were divided into four regions (or 27 for chr. 1) and averaged to evaluate the multifractality of each chromosome region.

### Molecular parameters and chaos game representation

The (G+C) contents and Ns were counted for each DNA fragment of 300 kb by a script written in Python. Likewise, several molecular parameters were counted from different files: CGI from seq_cpg_islands.gz file, Alu (Y, S, J), LINEs, MIRs, MERs and LTRs from seq_repeat.md.gz file, genes from seq_gene.md.gz file, exons and introns from gbk.gz file, SNPs from seq_snp.md.gz file, and the number of gene functions from rna.q file. All these files were downloaded from NCBI human build 36.2. KEGG(ftp://ftp.genome.jp/pub/kegg/pathway/organisms/hsa/), and KOGs (ftp://ftp.ncbi.nih.gov/pub/COG/KOG/) were analyzed. As control, we compared some molecular parameter profiles (G+C and Alus) with those reported in literature [2].

Subsequently, the CGR was implemented according to methods in [43,44]. Figure 1A shows an example of a CGR.

### Multifractal analysis and discrimination analyses

A fractal is a geometric fragmented figure whose parts are an approximate scaled copy of the whole figure, *i.e*., the figure possesses self-similarity. The fractal dimension *D *of the figure is basically the scaling rule the figure obeys. Generally, a power law is supposed:

$$N\left(E\right)\infty E^{-D}$$

where *N*(*E*) is the number of equal parts required to cover the figure when a scaling factor of *E *is applied. The power law allows to calculate the fractal dimension as

D=ln (N(E))/ln (E)

The fractal dimension obtained by the box-counting algorithm covers the figure with disjoint boxes of size ε = 1/*E *and counts the number of required boxes. Multifractal analysis is used, when multiple scaling rules apply. In this case, not one but a spectrum of fractal dimensions *D_q_*, for all integer *q*, are evaluated [24,44]. Generalizing the box-counting algorithm to the multifractal case, Eq. (1) is obtained:

(1) $$D_{q}\left(\varepsilon \right)=\frac{ln\left(\sum_{i}{\left(\frac{M_{i}}{M_{0}}\right)}^{q}\right)}{ln\left(\varepsilon \right)}\frac{1}{q-1}$$

where the number *M*_i _of points that fall in the *i*-th grid box is determined and related to the total number *M*_0 _and εis the box size.

The multifractal spectrum is obtained as the limit:

(2) $$D_{q}=\underset{\varepsilon \to \infty }{\lim}D_{q}\left(\varepsilon \right)$$

Variation of the integer *q *allows to emphasize different regions and discriminate their fractal behavior: Positive *q *values emphasize dense regions; a high *D_q _*stands for richness in structure and properties in these regions. Negative *q *values emphasize sparse regions; a high *D_q _*indicates much structure and properties in these regions. In real world applications, the limit *D_q _*is easily approximated from data by a linear fit: transformation of Eq. (1) yields

(3) $$\ln\left(M_{i}^{q}\right)=D_{q}\left(\varepsilon \right)\left(q-1\right)\ln\left(\varepsilon \right)+\left(q-1\right)\ln\left(M_{0}^{q}\right)$$

which shows that ln(*M_i_^q^*) for fixed *q *is a linear function in ln(ε), therefore *D_q _*can be evaluated as slope of the fitted relationship between ln(*M_i_^q^*) and (*q *- 1)ln(ε) [11]. We used this box-counting method for the multifractal spectrum estimation of CGR points and the corresponding analysis according to [10,45].

Directly from the multifractal dimension *D_q_*, the correlation exponent τ(*q*) is derived asτ(*q*) = (*q *-1)*D_q_*. The degree of multifractality, Δ*Dq*, is the difference between maximum and minimum values of *Dq*: Δ*Dq *= *Dq*max - *Dq*min [17,46]. When Δ*Dq *is high, the multifractal spectrum is rich in information and highly aperiodic; when Δ*Dq *is small; the resulting dimension spectrum is poor in information and highly periodic. For each chromosome the number of Alu versus the MD per fragment were plotted. Discrimination analyses were performed using 2-D and 3-D plots, with combined molecular and multifractal parameters.

### Statistics analyses

The whole data set and each set of chromosome fragments per chromosome were analyzed by simple and multivariate regressions using the PASW statistics 18 software, to determine the goodness of the fit of several molecular parameters versus MD [47]. For multivariate regression of Δ*Dq *the data were normalized (values between 0 and 1). In each chromosome we determined the 5 variables with highest coefficient absolute values and the most relevant ones were considered. For some molecular parameters, their RM at a 95% of occurrence level was analyzed. And to classify the human chromosomes, a clustering analysis was generated by using the Hierarchical Clustering Explorer version 3.5 program (HCE3.5) [48]. The clustering tree was generated by using the following parameters: row by row normalization by control, complete linkage method and Person's correlation coefficient.

## Abbreviations

CGR: Chaos Game Representation; MD or Δ*Dq*: multifractality degree; RM: range of multifractality; AMD: average of multifractality degree; LMM: low and medium multifractality; GCI: CpG islands; XCI: X chromosome inactivation; chr.: chromosome.

## Authors' information

**Pedro A. Moreno **was formerly a graduate student at University of Houston and is currently an assistant professor at the Universidad del Valle (http://eisc.univalle.edu.co). He teaches courses in bioinformatics, molecular biology, and information technologies. Pedro has participated in several researches working with biologists, mathematicians, and engineers in molecular biology, bioinformatics, metagenomics, fractal geometry and is currently an advisor for many students at the University. He pioneered bioinformatics research in Colombia with fractal geometry studies applied to biological problems. **Patricia E. Vélez **is a professor at Universidad del Cauca and Director of the BIMAC Group (http://bimac.unicauca.edu.co) and she has been pioneered in leadership several researches on breast cancer, human genetics, bioinformatics, and fractal geometry applied to genetics problems. **Ember Martínez **is a graduate student and professor at Universidad del Cauca. **Luis E. Garreta **is a doctoral student at Universidad del Valle. **Néstor M. Díaz **is a graduate student and professor at Universidad del Cauca. **Siler Amador **is a professor at Universidad del Cauca. **Irene Tischer **is a professor at Universidad del Valle and Director of the Laboratorio de Bioinformática. **José M. Gutiérrez **was formerly a post-doctoral associate at Cornell University and is currently a professor at Universidad de Cantabria, Spain. **Ashwinikumar K. Naik **is a medical doctor and bioinformatician in Bangalore and participated in the human genome sequencing in Celera Genomics. **Fabian Tobar **is a doctoral student and bioinformatician at Laboratorio de Bioinformática, Universidad del Valle. **Felipe Garcia **was formerly a postdoctoral associate at Harvard University. He is currently a professor at the Universidad del Valle and Director of the Laboratory of Molecular Biology and Microbiology.

## Authors' contributions

PAM conceived the research idea, participated in its design, execution, coordination, and drafted the manuscript. PEV helped to discuss, execute and draft the manuscript. EM carried out part of the writing of scripts in java for multifractal analysis and wrote part of the phyton code for molecular parameter counting. LG participated in setting up a bioinformatics server for processing and maintaining the databases. NMD and SA were responsible for security issues in informatics, web site technology (http://bimac.unicauca.edu.co) and mathematical approaches. IT was involved in the mathematics and statistical analyses and helped to discuss the manuscript. JMG verified the equations and java code for multifractal analysis. AKN helped to discuss the human genome characteristics. All previous authors are part of the research grant approved by COLCIENCIAS. FT and FG participated in the gene analyses. All authors read and approved the final manuscript.

## Supplementary Material

Additional file 1 **Multifractal and molecular parameters for whole human genome**. The file contains the Hs_refseq human genome sequence build 36.2 divided by fragments of 300 kb and multifractal and molecular parameters for 9,379 human chromosome fragments. All REs: All repetitive elements. Click here for file

Additional file 2 **τ(*q*) parameters for the human genome**. The file contains theτ(*q*) parameters for 9,379 human chromosome fragments. Click here for file

Additional file 3 **Multivariate analysis per chromosome**. The file contains a significant multivariate analysis per chromosome: consolidated components. Click here for file

Additional file 4 **Multivariate analysis for all genome**. The file contains a significant multivariate analysis for all genome. Click here for file

Additional file 5 **Determination coefficients per chromosome**. The file contains determination coefficients per chromosome between the MD *versus *several molecular parameters. NCFs: Number of chromosome fragments. NRCF: Number of removed chr. fragments. R^2 ^are indicated as R^2. The seventh column shows the corrected R^2 ^when nine data (sixth column) from the second column are removed from the analyses. (r): for 1 or 2 chromosomic fragments, few Alu content and high multifractality and (0): for 1 chromosomic fragment without Alu content. *: See Figure 3. All REs: All repetitive elements. Click here for file

Additional file 6 **VSTRs sequences**. The file contains chromosome fragment sequences with VSRTs for chromosomes 21 and Y. Click here for file

Additional file 7 **The most multifractal chromosome fragments in the human genome**. The file contains genomic location of the most multifractal chromosome fragments. Click here for file

Additional file 8 **Chromosome fragments with LMM**. The file contains a threshold definition and genomic location for the chromosome fragments with LMM. Click here for file

Additional file 9 **Gene function versus RM**. The file contains the analyses by gene functions, KEGGs, KOGs and exons versus RM. Click here for file

Additional file 10 **Gene family versus RM**. The file contains the analysis of gene family versus RM. Click here for file

Additional file 11 **Gene length versus RM**. The file contains the analysis of gene length versus RM. Click here for file

Additional file 12 **Averaged multifractality versus Alus**. The file contains averaged Δ*Dq *versus averaged Alus per chromosome. Click here for file

Additional file 13 **Averaged multifractality versus CGI**. The file contains averaged Δ*Dq *versus averaged CGI per chromosome. Click here for file

Additional file 14 **Multifractality and hierarchical clustering analysis**. The file contains the averaged multifractal parameters per chromosome for hierarchical clustering analysis. Click here for file

Additional file 15 **Chr 21 multifractal analysis by regions**. The file contains multifractal parameters by averaged region of chromosome 21. Click here for file

Additional file 16 **Chrs X and Y multifractal analyses by regions**. The file contains multifractal parameters by averaged region of chromosome X and Y. Click here for file

Additional file 17 **Chr 8 multifractal analysis by regions**. The file contains multifractal parameters by averaged region of chromosome 8. Click here for file

Additional file 18 **Chr 1 multifractal analysis by regions**. The file contains multifractal parameters by averaged region of chromosome 1. Click here for file

Additional file 19 **Chrs 4 and 19 multifractal analyses by regions**. The file contains multifractal parameters by averaged region of chromosomes 4 and 19. Click here for file

Additional file 20 **Averaged CGI versus multifractality of chr. 1**. The file contains averaged CGI versus averaged multifractality of chromosome 1. Click here for file
